# Population Dynamics of the Fiddler Crab *Tubuca rhizophorae* (Tweedie, 1950) in the Mekong Delta, Vietnam

**DOI:** 10.1002/ece3.71830

**Published:** 2025-07-23

**Authors:** Anh Ngoc Tran, Lam Thi Thao Vo, Quang Minh Dinh, Gieo Hoang Phan, Nhien Thi My Huynh, Bang Thi Nhu Le, Ton Huu Duc Nguyen

**Affiliations:** ^1^ Department of Biology, College of Natural Sciences Can Tho University Can Tho Vietnam; ^2^ Faculty of Biology Education, School of Education Can Tho University Can Tho Vietnam; ^3^ Faculty of Agriculture and Rural Development Kien Giang University An Giang Vietnam; ^4^ Faculty of Veterinary Medicine, College of Agriculture Can Tho University Can Tho Vietnam

**Keywords:** fiddler crab, growth, Mekong Delta, mortality, *Tubuca rhizophorae*

## Abstract

This study investigates the population characteristics of the fiddler crab, *Tubuca rhizophorae*, at two sites in the Mekong Delta, Vietnam. Monthly samples were collected from March 2024 to February 2025 in Dong Hai, Bac Lieu and Dam Doi, Ca Mau. Population parameters, including asymptotic carapace width (CW_∞_ = 23.18 mm), growth rate (*K* = 0.68/year), longevity (approximately 4.41 years), total mortality (3.22/year), natural mortality (1.55/year), fishing mortality (1.69/year), exploitation rate (0.52/year), and carapace width at first capture (CW_c_ = 13.60 mm), were estimated. The results indicate that the *T. rhizophorae* population exhibits rapid growth, relatively short longevity, and is subject to significant fishing pressure; the exploitation rate was determined to be 0.52/year, which exceeds the *E*_50_ value (0.401). The study also reveals two major annual recruitment peaks occurring at the beginning and end of the year. These findings provide critical baseline data for developing sustainable management strategies for this fiddler crab species, which are crucial for maintaining biodiversity and ecosystem health in the Mekong Delta.

## Graphical abstract

This study uses monthly field surveys and length‐frequency analysis to examine the population dynamics of the fiddler crab *Tubuca rhizophorae* in the Mekong Delta, Vietnam. Key parameters, including growth rate, mortality, and exploitation rate, were estimated, revealing that the species experiences rapid growth but is subject to significant fishing pressure, with exploitation rates exceeding sustainable thresholds. These findings provide essential data for conservation efforts and sustainable management strategies for *T. rhizophorae* in coastal ecosystems.

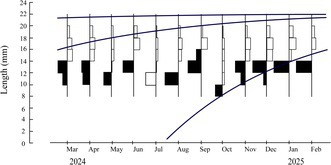

## 1 Introduction

Crustaceans play a crucial ecological and economic role in aquatic ecosystems, serving as key subjects for research to evaluate and manage fisheries resources sustainably (Smith and Addison 2003). Estimating growth, typically expressed through changes in body size, is a fundamental component in population dynamics modeling and in formulating sound management recommendations (Hoggarth 2006). In fisheries science, the von Bertalanffy Growth Function (VBGF) is a commonly used and significant model for illustrating how organism size correlates with age, offering valuable inputs for a range of stock evaluation models (Deriso 1980; Methot 1990; Quinn and Deriso 1999). Growth‐related parameters derived from the VBGF provide insights into population biological traits and are often associated with natural mortality rates (Beverton 1956; Pauly 1980). These metrics are frequently calculated using length‐at‐age datasets through nonlinear least squares approaches (Allen 1966; Kimura 1980; Seber and Wild 1989) and are extended results applied in fisheries resource management programs.

Despite their utility, growth studies in crustaceans pose unique methodological challenges. Unlike finfish, where growth assessment is relatively straightforward due to the availability of calcified structures such as otoliths, vertebrae, and scales for age determination (e.g., Summerfelt and Hall 1987; Smith 1992; Campana 2001), evaluating growth in crustaceans presents distinct challenges. This difficulty arises primarily because crustaceans periodically shed their exoskeleton through molting. Molting introduces two major complications: (1) potential aging structures are lost with each molt, making it impossible to directly determine age, and (2) growth occurs in irregular, stepwise increments rather than continuously. The absence of age‐specific anatomical markers prevents direct age estimation and, consequently, the acquisition of reliable size‐at‐age data. However, this limitation can be addressed using indirect methods such as length‐frequency analysis (Gulland and Rosenberg 1992) or by employing mark‐recapture techniques, where individuals are tagged and measured after a period of liberty to estimate growth based on changes in size (e.g., Wilder 1953; Hancock and Edwards 1967). Growth models such as VBGF can be applied without precise age data (Campbell and Phillips 1972).

From an ecological point of view, crustaceans' life‐history characteristics and growth are intimately related to their functions in ecosystems. Within coastal habitats such as mangrove forests, salt marshes, sandy beaches, and muddy flats, fiddler crabs (Ocypodidae: *Uca*) are one of the most conspicuous and easily observed species, and they often occur at higher densities than other brachyuran crabs inhabiting mangrove forests (Macia et al. 2001; Hartnoll et al. 2002; Skov et al. 2002). Fiddler crabs have a broad geographic distribution, ranging from tropical to warm temperate regions, and their species richness is peaking in the Americas, as noted in earlier studies (Crane 1975; Montague 1980). Their spatial distribution and zonation in intertidal habitats are strongly influenced by abiotic variables, including elevation gradients, salinity levels, temperature fluctuations, sediment composition, and organic matter availability (Gerlach 1958; MacNae 1968; Crane 1975; Icely and Jones 1978; Frith and Brunenmeister 1980). These fiddler crabs serve as critical ecosystem engineers, bridging energy transfer between trophic levels in mangrove and shallow‐water food webs. Research by Koch and Wolff (2002) highlights that detritivorous fiddler crabs, constituting over 95% of their population, contribute approximately 90% of the macrobenthic biomass in mangrove ecosystems. A substantial number of studies have explored their population dynamics and reproductive biology, focusing mainly on subtropical species (Colby 1984; Spivak et al. 1991; Emmerson 1994; Mouton and Felder 1995; Costa and Negreiros‐Fransozo 2002), whereas information on tropical species remains scarce (Litulo 2004a, 2004b).

Theoretically, crustacean life‐history strategies adapt to the selective pressures of extremely dynamic coastal environments. R‐selected traits, which promote colonization and persistence in uncertain environments, are generally associated with species that exhibit high reproductive output, short generation times, and rapid growth (Pianka 1970; Stearns 1998). These characteristics help detritivorous bioturbators, like fiddler crabs, adapt to changing environmental conditions. They also help them play the role of ecosystem engineers by improving nutrient cycling, organic matter decomposition, and sediment aeration. A further concern in fisheries‐induced evolution is that life‐history traits like high turnover and short lifespan may make an individual more susceptible to environmental stressors and anthropogenic disturbances, such as habitat degradation and harvesting pressure (Law 2007). As a result, knowledge of fiddler crab growth dynamics advances applied stock assessment and ecological theory by demonstrating how life‐history traits mediate species' ecological roles and responses to environmental change.

*Tubuca rhizophorae* (Tweedie 1950), belonging to the family Ocypodidae, typically occurs in high densities and is associated with elevated species richness and biomass within sandy‐muddy intertidal habitats (Rosenberg 2001; Skov et al. 2002). They play an essential ecological role in tropical and subtropical mangrove forests and represent a diverse group inhabiting various habitats (Bezerra and Matthews‐Cascon 2007). Additionally, they are valuable indicators for stock assessment across different fishing grounds and serve as a basis for biodiversity management and conservation strategies. In mangrove substrates, *T. rhizophorae* aids nutrient cycling and sediment mixing through burrowing and detritus feeding. Because of these ecological contributions, it is a valuable species for understanding material and energy flows in intertidal systems and monitoring fisheries. In Vietnam, 14 fiddler crab species have been recorded, with *T. rhizophorae* being one of the most commonly found in the Mekong Delta (Shih, Wong, et al. 2022). Although previous studies primarily focused on taxonomy and general morphology, *T. rhizophorae* is distinguished by its relatively flat carapace with less convergent lateral margins, small body size (maximum carapace width of ~20 mm), and a dark‐colored carapace in both sexes featuring a prominent marbled pattern with lighter tones ranging from olive green to yellow or light brown (Crane 1975; Shih, Prema, et al. 2022). However, there is currently limited information on the Ocypodidae species, particularly *T. rhizophorae*, in the coastal areas of the Mekong Delta. Moreover, critical population parameters—including size at first capture, maximum carapace width, longevity, growth coefficients (e.g., growth rate, natural mortality, fishing mortality), and exploitation thresholds (optimal, allowable, and maximum rates)—are poorly documented for this species.

To address these knowledge gaps, this study investigates the growth dynamics of *T. rhizophorae* populations in the Mekong Delta using length‐based VBGF modeling. In doing so, we aim to contribute to applied stock assessment and ecological theory regarding the life‐history strategies of detritivorous bioturbators in tropical intertidal systems. Specifically, this study addresses the following questions: (1) What are the key growth parameters (e.g., asymptotic size, growth rate) of *T. rhizophorae* in a tropical mangrove habitat? (2) How do these growth characteristics reflect its ecological role and susceptibility to environmental or anthropogenic pressures? We hypothesize that *T. rhizophorae* exhibits traits of an r‐selected strategist—such as fast growth and short lifespan—consistent with its role as a dominant benthic detritivore and ecosystem engineer in highly dynamic tropical intertidal environments.

## 2 Materials and Methods

### 2.1 Study Site and Sample Collection

**FIGURE 1:**
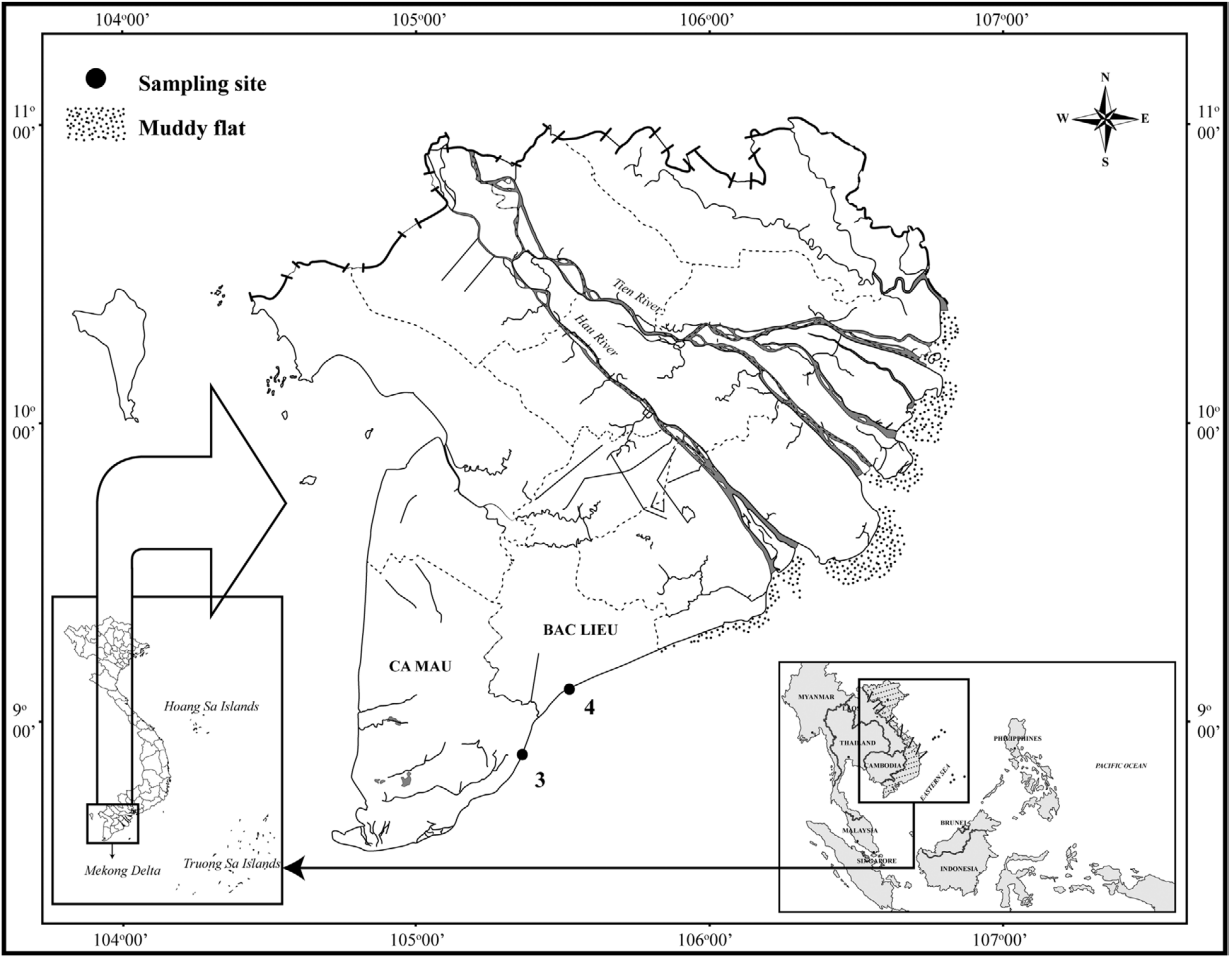
Map of sampling sites (•: Sampling site; 1: Dong Hai, Bac Lieu; 2: Dam Doi, Ca Mau).

Fiddler crab samples were collected monthly from March 2024 to February 2025 at Dong Hai, Bac Lieu, and Dam Doi, Ca Mau (Figure 1). The temperature, pH, and salinity recorded at the Dam Doi, Ca Mau, site were 30.46°C, 7.79, and 26.02‰, respectively, whereas the corresponding values at the Dong Hai, Bac Lieu, site were 30.94°C, 7.74, and 32.97‰. The typical plant species recorded at the two study sites included *Acanthus ebracteatus* Vahl., *Avicennia marina* (Forssk.) Vierh., *Bruguiera gymnorrhiza* (L.), *Nypa fruticans* Wurmb., *Sonneratia caseolaris* (L.) A. Engl., and *Rhizophora apiculata* Blume. Among them, *B. gymnorrhiza* was the dominant species at Dong Hai, Bac Lieu, whereas both *A. marina* and *B. gymnorrhiza* were co‐dominant at Dam Doi, Ca Mau (Tran and Dinh 2025). Fiddler crabs were collected monthly at low tide during daylight hours using manual hand capture and passive baited traps placed in representative microhabitats within the intertidal zones. At each sampling site, three sampling plots were selected; each plot was ~15 m^2^ (3 m wide × 5 m long) and spaced 5 m apart. Each sampling time lasted roughly 2–3 h per site, covering all plots. Individuals were selectively collected to represent all observable size classes, including juveniles and adults, to minimize potential bias in carapace width distribution. After collection, specimens were fixed in 70% ethanol, labeled, and transported to the laboratory for species identification and morphometric analysis.

**FIGURE 2:**
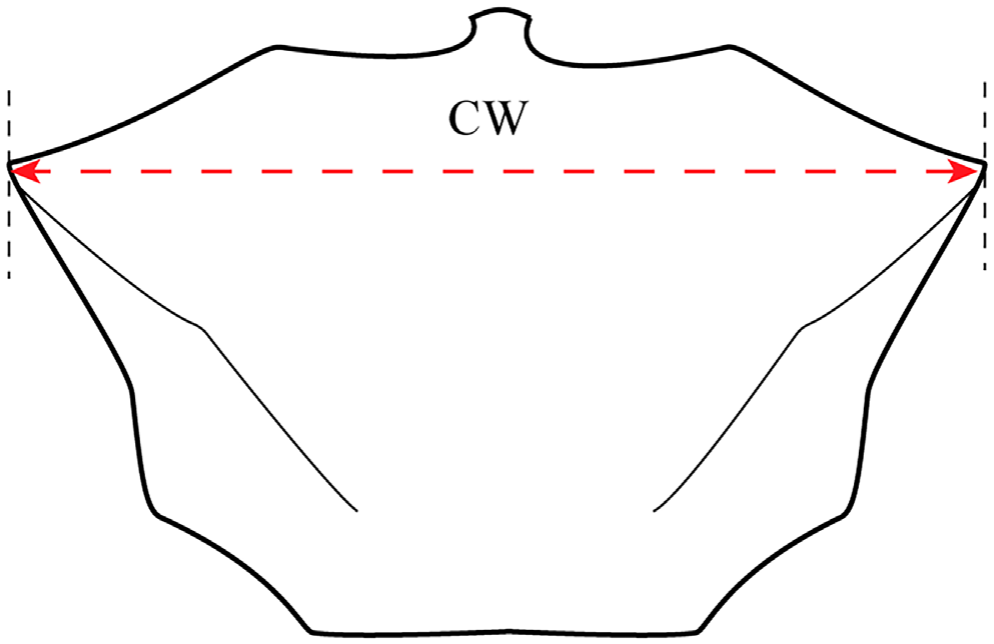
Illustration of carapace width (CW) of *Tubuca rhizophorae*.

Fiddler crab specimens were identified in the laboratory using morphological characteristics outlined by Tweedie (1950). The specimens were then measured for carapace width (CW) using an electronic digital caliper (accurate to 0.01 mm) (Figure 2).

### 2.2 Data Analysis

Population parameters were estimated from length‐frequency data using von Bertalanffy (1938): ${\mathrm{CW}}_{t}={\mathrm{CW}}_{\infty }\times \left[1-e^{-K\left(t-t_{0}\right)}\right]$, where CW_∞_ is the asymptotic carapace width (mm) that individuals can theoretically attain; *K* is the growth rate describing the rate at which CW_∞_ is approached; *t* is the age of the crab at time *t*; and *t*_0_ is the theoretical age at which the carapace width would be zero. These growth parameters are ecologically meaningful, reflecting species' life‐history strategies—whether they prioritize rapid growth and short lifespans (*r*‐selected traits) or slow growth and competitive dominance (*K*‐selected traits).

The CW frequency data of males and females were pooled together because size by sex was insufficient for analysis. The original carapace width frequency data were input into the FiSAT II software to estimate the initial CW_∞_ using the Powell‐Wetherall procedure (Powell 1979; Pauly 1986b; Wetherall 1986). The initial CW_∞_ value was then used to estimate the *K* index was estimated using the ELEFAN I routine within the FISAT II software. This involved accessing the “Direct fit of F/L data” function, followed by executing the “ELEFAN UK Scan/Compute” command (Gayanilo et al. 2005). The resulting CW_∞_ and *K* estimates were then used to develop capture probability curves. These curves facilitated carapace width frequency data adjustment, addressing capture selectivity by including CW_25_, CW_50_, and CW_75_ (Pauly 1986a). The adjusted frequency data were subsequently used to refine the final CW_∞_ and *K* estimates via the ELEFAN I method (Pauly and David 1981).

The growth coefficient index (Φ′) was estimated using the formula Φ′ = log *K* + 2 × log CW_∞_, proposed by Pauly and Munro (1984). This composite metric allows comparison of overall growth efficiency across species and populations and may reflect adaptations to local environmental constraints such as temperature, food availability, and predation pressure.

The longevity (*t*_max_) of the crabs was calculated according to the following formula: *t*_max_ = *t*_0_ − (1/*K*)ln[1 − (CW_*t*_/CW_∞_)], where *K* is the growth parameter; CW_∞_ is the asymptotic carapace width (mm) that crabs can theoretically attain; and CW_*t*_ is the estimated mean CW at age *t*. From an ecological standpoint, longevity is a key determinant of reproductive potential and turnover rates, affecting detrital processing and nutrient cycling in benthic systems.

The total mortality rate (*Z*) was determined from length‐converted catch curves using length‐frequency data, with *Z* = *F* + *M* (Pauly et al. 1995). The natural mortality rate (*M*), which accounts for mortality due to adverse environmental conditions, diseases, or predation during the natural life cycle, was estimated using the empirical equation of Pauly (1980): Ln(*M*) = −0.0152–0.279 × ln(CW_∞_) + 0.6453 × ln(*K*) + 0.463 × ln(*t*), where CW_∞_ is the asymptotic carapace width (mm); *K* is the growth parameter; *T* is the mean annual water temperature (°C). Natural mortality reflects the population's exposure to environmental stressors, predation, and disease, integral to ecological resilience.

The fishing mortality rate (*F*), representing mortality caused directly or indirectly by fishing activities, and the exploitation rate (*E*) were calculated according to the formula *F* = *Z*–*M* and *E* = *F*/*Z* provided by Ricker (1975). The exploitation rate (*E*) is ecologically relevant because it indicates whether the population is being harvested sustainably or is at risk of overexploitation, with potential cascading effects on food web dynamics and sediment bioturbation.

The length at first capture (CW_c_), which is defined as the carapace width at which 50% of individuals are vulnerable to fishing, was determined using the length‐converted catch curve method (Pauly 1987). The Beverton and Holt (1957) yield‐per‐recruit (*Y*′/*R*) and biomass‐per‐recruit (*B*′/*R*) models were applied to estimate the exploitation rate for maximum yield (*E*_max_), the exploitation rate at which *Y*′/*R* increases by 10% (also referred to as the optimal exploitation rate, *E*_10_), and the exploitation rate at which *B*′/*R* is reduced by 50% (*E*_50_). Additionally, the ratio of CW_c_ to CW_∞_ (the exploitation isopleth ratio) was also analyzed. The CW_c_/CW_∞_ ratio combined with the estimated exploitation rate (*E*) was used to assess the exploitation status of the crab population, following the approach of Pauly and Soriano (1986). These parameters provide insight into the species' capacity to withstand anthropogenic pressures and maintain its functional role in intertidal ecosystems.

## 3 Results

**FIGURE 3:**
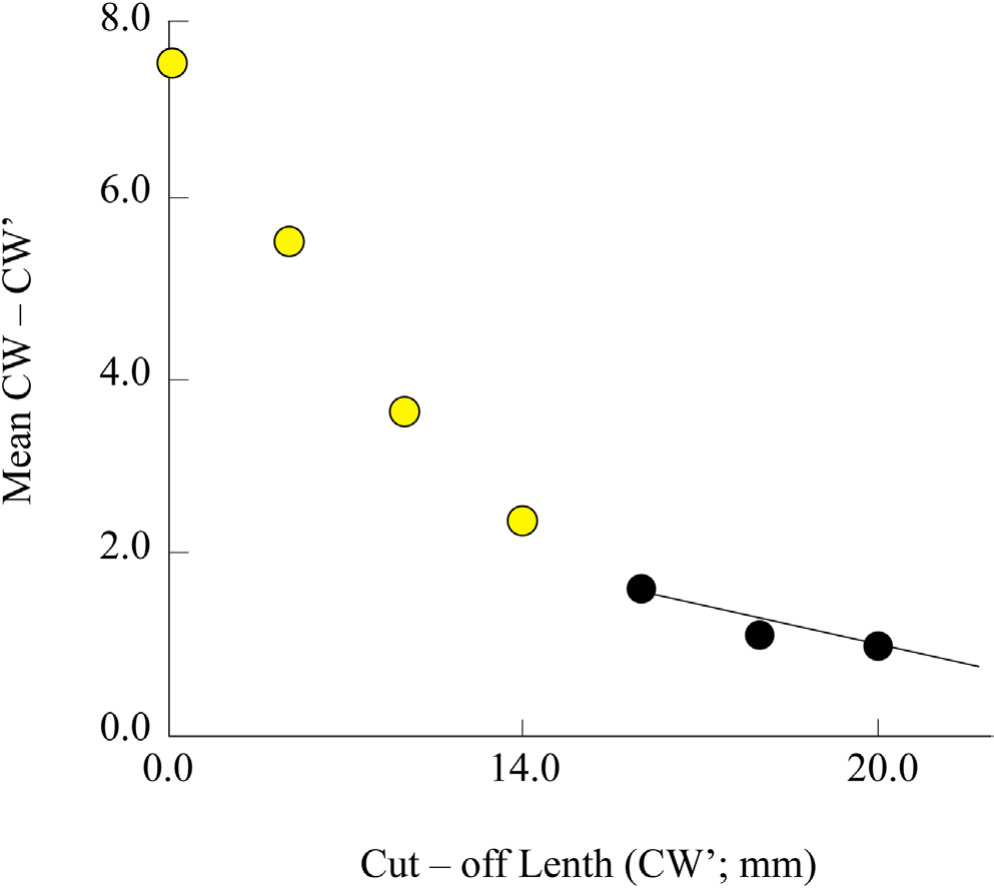
Powell‐Wetherall plots of *Tubuca rhizophorae* (CW′: Cut off carapace in mm; Yellow data points: Not used; Dark data points: Used).

A total of 760 *T. rhizophorae* specimens (267 females and 493 males) were collected from the two study sites over 12 months. The species' mean carapace width and body weight were recorded as 15.53 ± 0.07 mm and 1.58 ± 0.02 g (SE), respectively. The mean value of CW–CW′ was 16.00 mm, and CW′ was 1.60 mm. Model estimates indicated that CW_∞_ was 23.18 mm, and the *Z*/*K* ratio was 3.49, reflecting the relationship between total mortality and the species' growth rate. The linear regression equation was determined as *Y* = 5.16–0.223*X*, with a correlation coefficient (*r*) of −0.98, demonstrating a strong alignment between the observed data and the model. Additionally, the *Z*/*K* value greater than 3 suggests that the total mortality rate of the population was relatively high compared to its natural growth rate (Figure 3).

**FIGURE 4:**
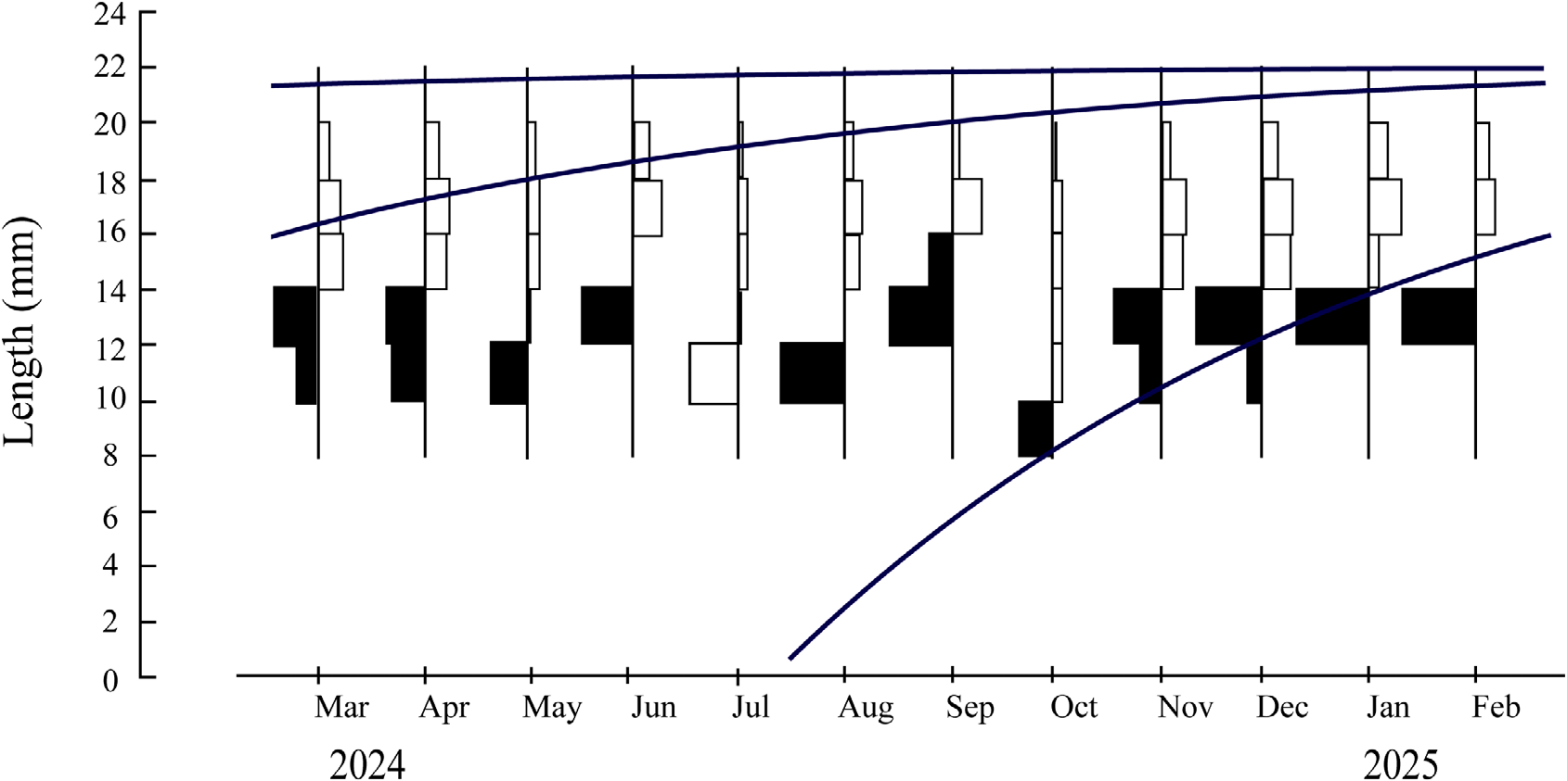
Growth curves of *Tubuca rhizophorae* estimated using ELEFAN I superimposed on restructured corrected carapace width‐frequency data (three curves represented for three cohorts in the population).

The carapace width (CW) frequency data of *T. rhizophorae* from the study area were analyzed using the von Bertalanffy Growth Model, with the results presented in Figure 4. The figure illustrates monthly variations in CW class frequency distributions, represented by black and white bars, along with overlaid growth curves showing the temporal progression of CW. The individuals with smaller carapace widths exhibited a faster growth trend, as evidenced by the steeper slope of the growth curve during the early stages. In contrast, the group of individuals with larger sizes displayed a slower growth trend as their carapace width approached the asymptotic value (CW_∞_). The analysis revealed that the *T. rhizophorae* population in the study area was characterized by two superimposed growth cohorts, as interpreted from the CW frequency data. The von Bertalanffy model parameters were estimated as CW_∞_ = 23.18 mm, *K* = 0.68/year, and *t*_0_ = −0.25, resulting in the von Bertalanffy growth equation for the population: CW_*t*_ = 23.18 (1 – *e* ^−0.68(*t* + 0.25)^). This analysis indicates that *T. rhizophorae* exhibited a fast growth rate, consistent with the ecological traits and short life cycle of this crab species in the studied mangrove habitat.

**FIGURE 5:**
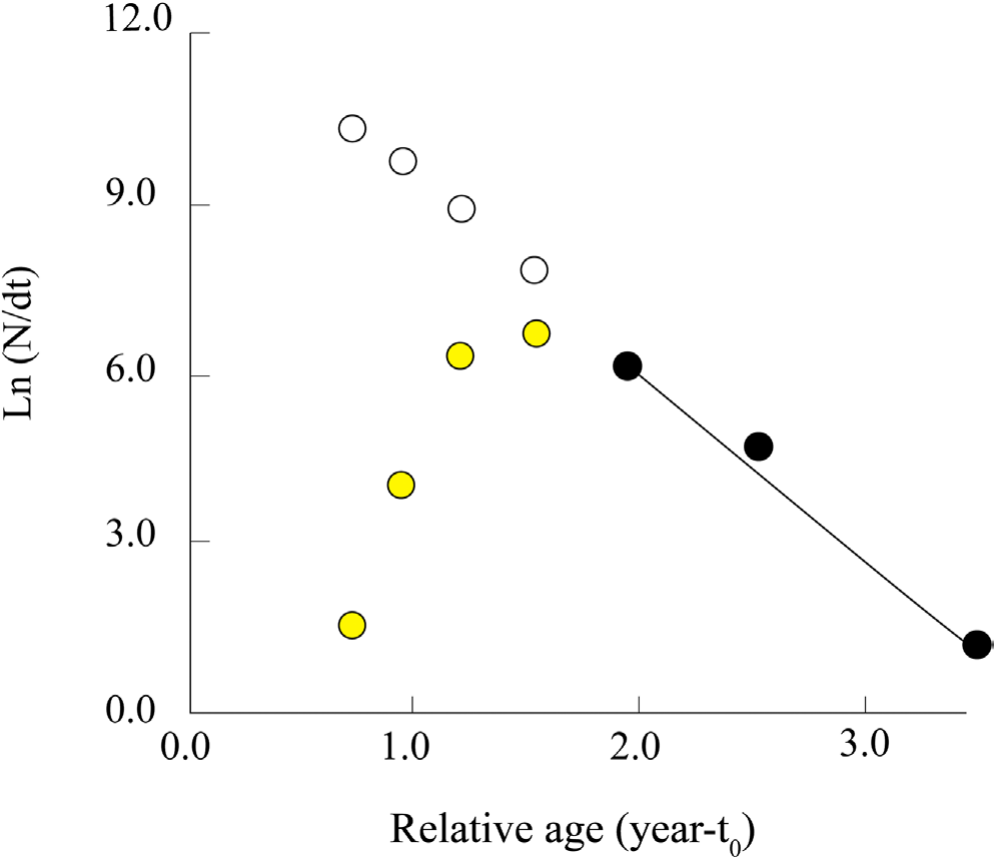
Catch curve based on carapace width for *Tubuca rhizophorae* (*Z* = 3.22 year^−1^, *M* = 1.55 year^−1^, *F* = 1.69 year^−1^, *E* = 0.52; Yellow data points: Excluded; Dark data points: Excluded; Open circles represent predicted abundance in partially selected size classes).

The carapace width frequency data yield curve showed that *Z* = 3.22/year, *M* = 1.55/year, *F* = 1.69/year, and *E* = 0.52/year (Figure 5).

**FIGURE 6:**
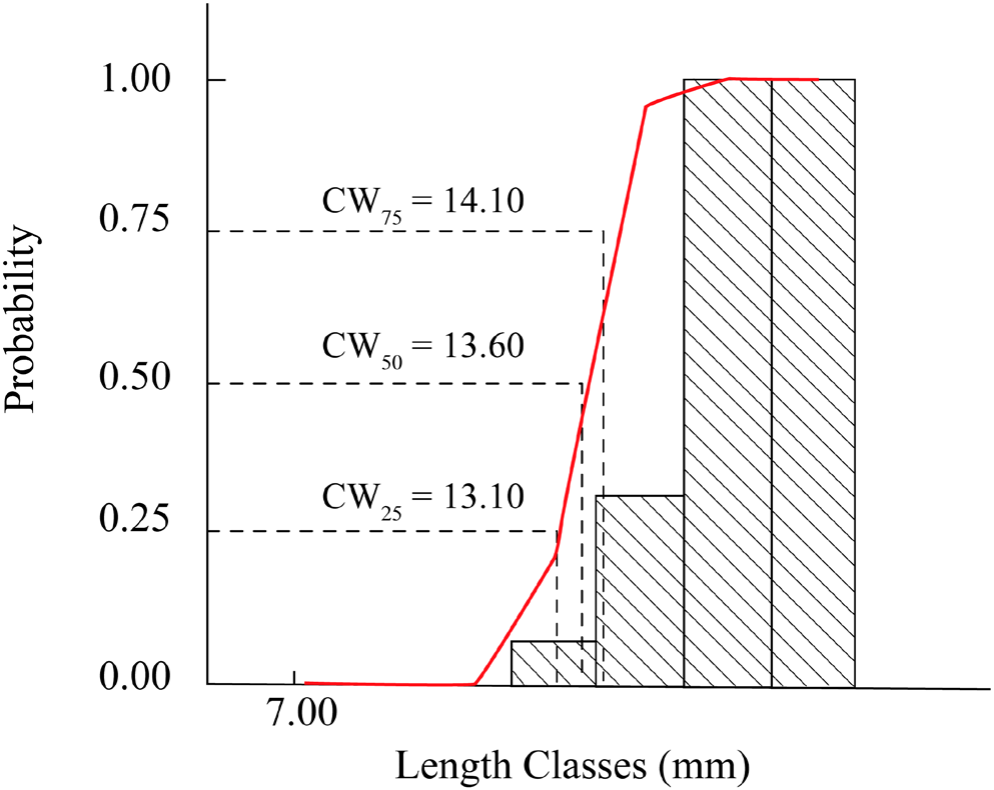
Probability of capture based on carapace width of *Tubuca rhizophorae* with CW_25_, CW_50_, and CW_75_ thresholds.

The analysis revealed that the CW_c_ (or CW_50_) was 13.60 mm. Meanwhile, CW_25_ and CW_75_ were estimated at 13.10 and 14.10 mm, respectively (Figure 6). This indicated that individuals smaller than 13.10 mm displayed a low probability of being captured (below 25%), whereas those exceeding 14.10 mm exhibited a capture probability greater than 75%.

**FIGURE 7:**
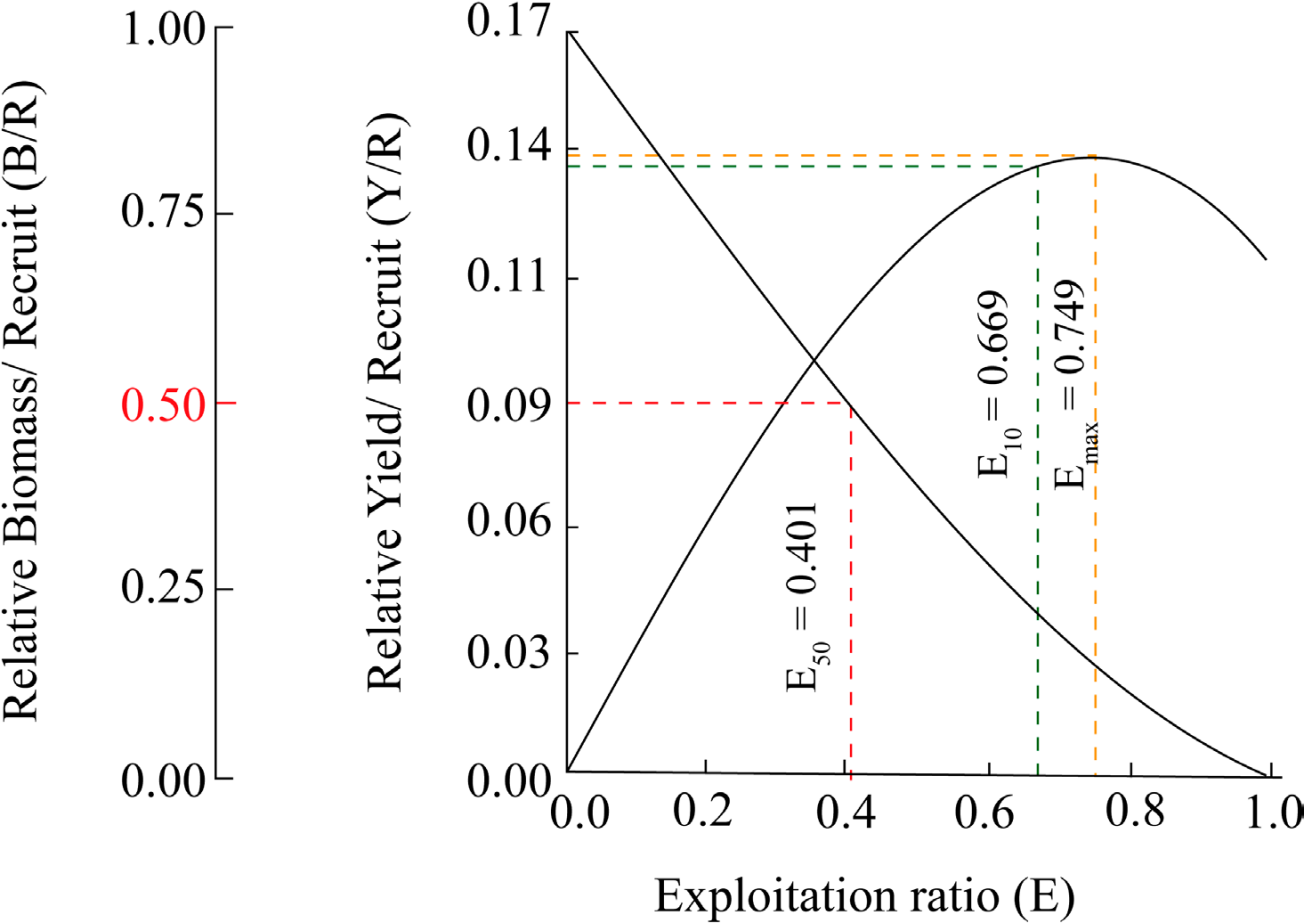
The relative yield‐per‐recruit (solid curve) and relative biomass‐per‐recruit (broken curve) using the knife‐edge selection procedure of *Tubuca rhizophorae*.

**FIGURE 8:**
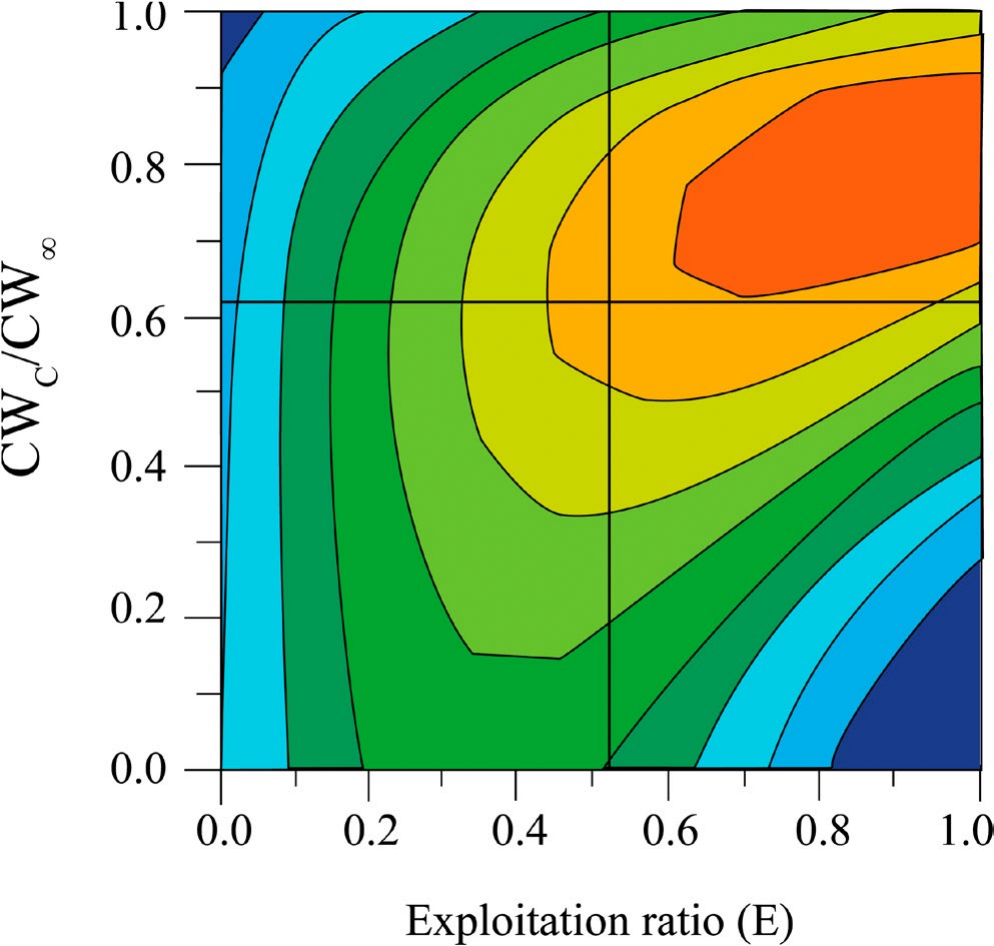
The relative yield‐per‐recruit isopleth diagram of *Tubuca rhizophorae* (CW_c_/CW_∞_ = 0.617, CW_c_ = 13.6 mm, CW_∞_ = 23.18 mm, *E* = 0.52).

Yield‐per‐recruit and biomass‐per‐recruit analyses for the *T. rhizophorae* population in the study area estimated *E*_max_, *E*_10_, and *E*_50_ at 0.749, 0.669, and 0.401, respectively (Figure 7). The CW_c_/CW_∞_ and *M*/*K* ratios were 0.62 and 0.73, respectively, indicating a moderate level of exploitation for this population (Figure 8). The growth coefficient index (Φ′) for the *T. rhizophorae*, derived using the formula Φ′ = log *K* + 2 × log CW_∞_, was 2.56. Additionally, the maximum estimated longevity of the population was approximately 4.41 years.

**FIGURE 9:**
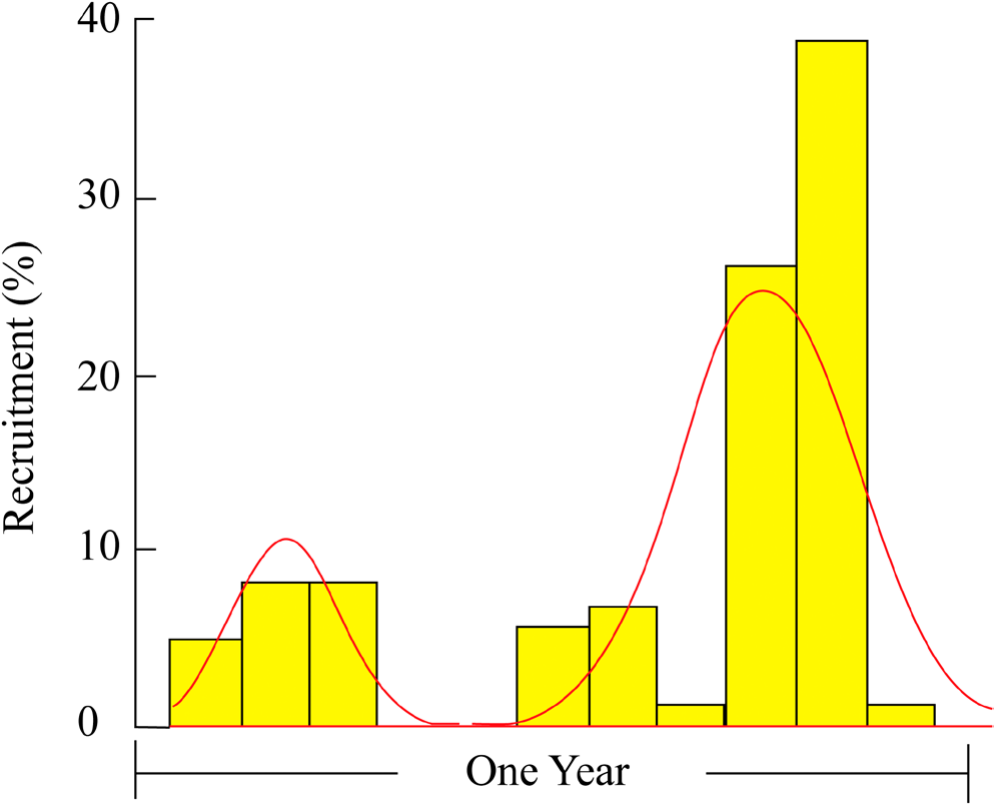
Annual recruitment pattern (%) of the *Tubuca rhizophorae* population at the study site.

Figure 9 illustrates the annual recruitment pattern of the *T. rhizophorae* population in the study area, revealing two major recruitment pulses per year. The first recruitment event occurred around Month 2 (early in the year), whereas the second happened around Month 9 (late in the year). The bar chart showed the percentage of individuals entering the population each month, with the highest peak observed during the second recruitment event. These results indicated that *T. rhizophorae* exhibits year‐round reproduction, with two prominent peaks, reflecting a reproductive strategy adapted to the environmental conditions of the study area.

## 4 Discussion

Our research identified key population parameters of *Tubuca rhizophorae* in the study area, with a CW_∞_ of 23.18 mm, a *K* of 0.68 per year, and a *t*_max_ of ~4.41 years. The exploitation rate of 0.52 year^−1^ exceeded the optimal exploitation rate (0.401), indicating that the population is currently subject to overexploitation. This result highlights the urgent necessity of implementing effective management strategies to ensure the long‐term sustainability of the *T. rhizophorae* population. When comparing these growth parameters with those reported for other Ocypodidae species, distinct similarities and differences are evident, attributable primarily to environmental variation, food resource availability, and species‐specific life history strategies. For instance, Lavajoo et al. (2014) documented a notably higher growth rate (*K* = 1.8 year^−1^) and exploitation rate (*E* = 0.68) for *Uca sindensis* (Alcock, 1900) inhabiting the mangrove forests of Pohl in the Persian Gulf, Iran, compared to our findings for *T. rhizophorae*. Similarly, Koch et al. (2005) reported substantially greater *K* values, ranging from 2.03 to 4.24 year^−1^, for four *Uca* species— *Uca cumulanta* Crane (1943), *Uca maracoani* Oliveira (1939), *Uca rapax* (Smith, 1870), and *Uca vocator* (Herbst, 1804)—within the mangrove estuaries of Northern Brazil. These elevated growth rates are characteristic of species or populations thriving in highly productive tropical environments with abundant and stable food supplies year‐round and typically reflect a fast‐growing, short‐lived life history strategy (Masunari 2006). Indeed, Koch et al. (2005) also observed that these four *Uca* species exhibited rapid growth and relatively short maximum lifespans (ranging from 0.7 to 1.47 years), consistent with their comparatively smaller asymptotic carapace widths (CW∞): *Uca cumulanta* (13.1 mm for males and 11.1 mm for females), *Uca maracoani* (35.2 and 31.0 mm), *Uca rapax* (20.5 and 20.0 mm), and *Uca vocator* (21.6 and 20.6 mm). Conversely, our *K* value (0.68 year^−1^) for *T. rhizophorae* is higher than those reported by da Silva‐Castiglioni and Negreiros‐Fransozo (2004) for *U. rapax* (*K* = 0.21 year^−1^ for males and 0.16 year^−1^ for females). Such disparities in growth rates and sizes at sexual maturity among crustacean species can be influenced by a complex interplay of environmental factors (Teissier 1960; Campbell and Eagles 1983; von Hagen 1987). Key abiotic factors, including temperature, photoperiod, rainfall, and the availability and quality of food resources, directly impact metabolic rates and growth efficiency. Tropical regions, characterized by less extreme seasonal fluctuations and consistently high temperatures throughout the year, often facilitate continuous and faster growth rates compared to subtropical or temperate zones due to the stable availability of food (Masunari 2006).

The estimated natural mortality rate (*M*) of *T. rhizophorae* was 1.55 year^−1^, which is lower than the total mortality rate (*Z*) of 3.22 year^−1^, indicating that fishing mortality (*F*) contributes significantly (1.69 year^−1^) to overall mortality. The *M*/*K* ratio of 0.729 suggests a moderate level of exploitation. The carapace width at first capture (CW_c_) was 13.60 mm, equivalent to 58.7% of CW_∞_. This implies that *T. rhizophorae* is being harvested before reaching its maximum adult size, which can negatively impact the population's reproductive potential and long‐term sustainability. Comparing total mortality, Koch et al. (2005) reported considerably higher *Z* values for all four *Uca* species they studied (ranging from 4.6 to 7.6/year), with the highest values observed in male and female *U. cumulanta* (9.1 and 10.6/year, respectively). These extremely high total mortality rates, often exceeding fishing mortality, suggest that predation can be the most significant cause of mortality for fiddler crabs (Wolff et al. 2000; Koch and Wolff 2002). Fiddler crabs are indeed a crucial dietary component for numerous predators, including birds, fish, predatory crabs, and mammals (Montague 1980; Montague et al. 1981; Jones 1984; Wiedemeyer 1997; Keuthen 1998; Wolff et al. 2000).

According to eco‐evolutionary theory, harvesting individuals before they reach reproductive maturity may impose directional selection on life‐history traits, favoring smaller adult body sizes and earlier maturation. Long‐term changes in population structure and adaptive potential can result from these selective pressures, which are commonly known as fisheries‐induced evolution (Law 2007). *T. rhizophorae*'s moderate lifespan and observed rapid growth point to a primarily r‐selected strategy (Pianka 1970), which allows for persistence in dynamic intertidal environments. These characteristics, though, might also make individuals more sensitive to human disturbances. In this regard, mangrove ecosystems' spatial refugia or protected enclaves may serve as evolutionary buffers. These regions preserve life‐history variation and adaptive capacity by protecting specific populations from severe harvest pressure, conserving phenotypic and genotypic diversity (Yosef et al. 2024). These refuges are crucial for maintaining evolutionary resilience and reducing harvest‐driven directional selection. These results highlight the necessity of using an integrated ecological and evolutionary framework to interpret population‐level metrics, such as growth rates and exploitation ratios. This method advances general theory on how detritivorous ecosystem engineers react to selection gradients in environments that humans have altered, going beyond applied stock assessment.

Our recruitment analysis revealed two distinct recruitment pulses per year for the *T. rhizophorae* population, occurring at the beginning and end of the year. This pattern aligns with the reproductive strategies of many fiddler crab species, which often release their larvae and subsequently recruit to exploit favorable environmental conditions for optimal juvenile survival. The high population turnover rate and productivity of fiddler crabs explain their pivotal role in intertidal food webs, where they efficiently convert microbial production into energy for higher trophic levels (e.g., Teal 1962; Montague 1980; Koch and Wolff 2002). In comparison, Litulo (2005a) found that recruitment in fiddler crab populations generally occurs year‐round, evidenced by the continuous presence of smaller individuals throughout the study period. Specifically, in this study, recruitment was strongest during the winter (April–August) and decreased in the summer (December–March). Juveniles recruited in winter could potentially grow and attain sexual maturity by late winter and early summer, when the presence of newly recruited juveniles was low, a pattern also noted by Emmerson (1994) and Gonzalez Acosta et al. (2004). Similarly, several studies on temperate and subtropical *Uca* populations, such as *Uca spinicarpa* Rathbun, 1900 (Mouton and Felder 1995), *Uca subcylindrica* (Stimpson, 1859) (Rabalais and Cameron 1983), *Uca uruguayensis* (Nobili, 1901) (Spivak et al. 1991), *Uca subcylindrica* (Thurman 1985), and *Uca lactea* (De Haan, 1835) (Yamaguchi 2001), often exhibit bimodal size distributions, a characteristic associated with distinct seasonal spawning periods. Conversely, some studies on tropical fiddler crab populations, such as *Uca thayeri* Rathbun, 1900 (de Almeida Farias et al. 2014), *U. inversa* (Hoffmann, 1874) (Litulo 2005a), and *U. urvillei* (H. Milne Edwards, 1852) (Litulo 2005b) tend to exhibit a unimodal pattern. However, unimodal recruitment has also been reported in other regions for species such as *U. vocator* (Colpo and Negreiros‐Fransozo 2004), *U. rapax* (Castiglioni and Negreiros‐Fransozo 2006), and various *Uca* populations (Bedê et al. 2008). Unimodal patterns are typically characteristic of stable populations where recruitment and mortality rates remain consistent throughout the life cycle, and the number of individuals entering the population is similar to those leaving (Thurman 1985; Díaz and Conde 1989). Often, peaks in reproduction are linked to fluctuations in environmental factors such as temperature, salinity, oxygen, food availability, photoperiod, and rainfall (Meusy and Payen 1988; Costa and Negreiros‐Fransozo 2002; Pinheiro and Fransozo 2002; Mantelatto et al. 2003; Litulo 2004b). Local environmental conditions likely shape the two main recruitment pulses we observed and reflect the species' adaptive reproductive strategy for maximizing survival in the fluctuating intertidal zones of the Mekong Delta.

## 5 Conclusion

This study highlights the overexploitation of *T. rhizophorae* in the Mekong Delta, a species vital for mangrove ecosystem health. With exploitation rates exceeding sustainable levels and premature harvesting compromising reproductive potential, immediate management interventions are crucial. The identified recruitment peaks provide key opportunities for seasonal protection. We recommend implementing size limits, seasonal closures, and catch quotas to safeguard this resource. Protected areas or spatial refugia should also be established to maintain natural variation in life‐history traits and safeguard against harvest‐driven selection. The results support the inclusion of life‐history and eco‐evolutionary theories in conservation strategies, particularly for detritivores subject to variable and artificial stressors. An ecosystem‐based approach, integrating community engagement and habitat restoration, is essential for ensuring the long‐term resilience of *T. rhizophorae* and the ecological integrity of the Mekong Delta.

## Author Contributions

**Anh Ngoc Tran:** investigation (equal), methodology (equal), writing – original draft (equal), writing – review and editing (equal). **Lam Thi Thao Vo:** investigation (equal), methodology (equal), writing – original draft (equal), writing – review and editing (equal). **Quang Minh Dinh:** investigation (equal), methodology (equal), supervision (equal), writing – original draft (equal), writing – review and editing (equal). **Gieo Hoang Phan:** investigation (equal), methodology (equal), supervision (equal), writing – original draft (equal), writing – review and editing (equal). **Nhien Thi My Huynh:** investigation (equal), methodology (equal), writing – original draft (equal), writing – review and editing (equal). **Bang Thi Nhu Le:** investigation (equal), methodology (equal), writing – original draft (equal), writing – review and editing (equal). **Ton Huu Duc Nguyen:** methodology (equal), visualization (equal), writing – original draft (equal), writing – review and editing (equal).

## Ethics Statement

The authors have nothing to report.

## Conflicts of Interest

The authors declare no conflicts of interest.

## Supporting information


Data S1.

## Data Availability

Data were uploaded to the journal system as Supporting Information for review and publication.
